# Shift work is significantly and positively associated with possible gastro-esophageal reflux disease: A meta-analysis study

**DOI:** 10.3389/fpubh.2022.980603

**Published:** 2022-11-25

**Authors:** Hsiang-Tai Chen, Hung-Yi Chuang, Tsung-Yu Hsieh, Pei-Shan Wu, Fang-Jiun Lin, Huan-Chih Huang, Chen-Cheng Yang, Chao-Hung Kuo

**Affiliations:** ^1^College of Health and Medicine, University of Tasmania, Hobart, TAS, Australia; ^2^Department of Occupational and Environmental Medicine, Kaohsiung Medical University Hospital, Kaohsiung Medical University, Kaohsiung City, Taiwan; ^3^Department of Public Health and Environmental Medicine, and Research Center for Environmental Medicine, Kaohsiung Medical University, Kaohsiung City, Taiwan; ^4^Department of Occupational and Environmental Medicine, Kaohsiung Municipal Siaogang Hospital, Kaohsiung Medical University, Kaohsiung City, Taiwan; ^5^Department of Human Resource, Kaohsiung Municipal Siaogang Hospital, Kaohsiung Medical University, Kaohsiung City, Taiwan; ^6^Department of Family Medicine, Kaohsiung Municipal Siaogang Hospital, Kaohsiung Medical University, Kaohsiung City, Taiwan; ^7^Research Center for Precision Environmental Medicine, Kaohsiung Medical University, Kaohsiung City, Taiwan; ^8^Department of Internal Medicine, Kaohsiung Municipal Siaogang Hospital, Kaohsiung Medical University, Kaohsiung City, Taiwan

**Keywords:** shift work, gastro-esophageal reflux disease (GERD), occupational medicine (MeSH), meta-analysis, circadian rhythm

## Abstract

**Background:**

One of the health issues related to shift work patterns is possible gastro-esophageal reflux disease (GERD) symptoms. However, the association between shift work and possible GERD symptoms through meta-analysis has not been developed in the current literature field. Therefore, the purpose of this study is to analyze the association between shift work and possible GERD symptoms through meta-analysis.

**Methods:**

Studies containing target keywords were found in three datasets, and four articles were selected for further analysis after examining the title, abstract, and text. All prevalence odds ratios (ORs) among different groups of the population and the standard error (SE) from each included study were calculated for conducting meta-analysis.

**Result:**

The pooled OR has shown a significant positive association between shift work and possible GERD (OR 1.53; 95% confidence interval [CI] 1.33–1.77, *p*-value 0.003). Compared to non-shift workers, the subgroup analysis indicates there are positive associations between possible GERD and the night shift (OR 1.39; 95% CI 1.16–1.66), and the rotating shift (OR 1.83; 95% CI 1.44–2.33). The subgroup analysis has also shown similar trends in shift working men (OR 1.28; 95% CI 1.03–1.60) and shift workers of both genders (OR 1.75; 95% CI 1.45–2.11).

**Conclusion:**

This study has shown a positive association between shift work and possible GERD.

## Introduction

Shift work patterns have become common nowadays due to the round-the-clock activities (1). There are different types of shift work, including night work, rotating shifts, or irregular schedules. According to the data from the U.S. Bureau of Labor Statics in the period of 2017–2018, around 145 million (16%) workers work under a non-daytime schedule or by shift (2). However, shift work pattern is related to circadian rhythm disruption and that is associated to higher risk of some health issues, such as type 2 diabetes mellitus (3), obesity (4), and cardiovascular disease (5), also it has an impact on cognitive vulnerability (6) and mental health (7). Consequently, shift work has been discussed more frequently in recent years, because of its massive influence on health (8).

The gastrointestinal system plays a vital role in our health, and gastroesophageal reflux disease (GERD) is a common medical complaint. According to the World Gastroenterology Organization Global Guidelines, patients who have gastrointestinal symptoms two or more times per week and symptom relief after taking acid-suppressive medications match the diagnosis of GERD (9). In 2017 the prevalence of GERD was about 14% around the world, which estimates that about 1 billion people suffered from GERD. Several risk factors associated with GERD have been identified such as gender, obesity, and intake of coffee or tea (10). As well, some medicines (non-steroidal anti-inflammatory drugs, acetylsalicylic acid, anti-cholinergics, and theophylline) were also associated with the development of GERD (11). Medication management of GERD includes antacid, histamine 2-receptor antagonists, and proton pump inhibitors (PPI). Laparoscopic anti-reflux surgery may be indicated for structural disease (hiatus hernia) or persistent nocturnal symptoms despite PPI use (9, 12).

Previous studies have examined the association between shift work and gastrointestinal disorders, such as colorectal cancer or inflammatory bowel disease (13–15). However, relatively few studies have been published on the relationship between shift work and GERD (13), and a meta-analysis of the relationship is not available till now. Therefore, this study aims to analyze the association between shift work and possible GERD through meta-analysis.

## Materials and methods

### Protocol and registration

The meta-analysis has been implemented on the basis of the Preferred Reporting Items for Systematic Reviews and Meta-Analyses (PRISMA) guidelines. A search of electronic databases was performed, the titles, abstracts, and full text of returned papers were screened by independent researchers. Any conflict between studies was resolved between researchers. The review protocol has been registered at PROSPERO (ID CRD42022302196) and Kaohsiung Medical University Hospital Institutional Review Broad (KMUHIRB-EXEMPT(I)-20220006).

### Data sources and search terms

All studies containing the specific search terms were found in three datasets, PubMed, Embase, and Web of Science on 12 April 2022, and no limitation of publication dates has applied in the selecting process.

The keywords used as search terms in the preliminary search by researchers (T-YH and C-CY) were as follows: (((“shift”[All Fields] OR “shifted”[All Fields] OR “shifting”[All Fields] OR “shiftings”[All Fields] OR “shifts”[All Fields]) AND (“work”[MeSH Terms] OR “work”[All Fields])) OR (“Shift Work Schedule”[All Fields] OR “schedule shift work”[All Fields] OR “schedules shift work”[All Fields] OR “work schedule shift”[All Fields] OR “Night Shift Work”[All Fields] OR “shift work night”[All Fields] OR “Rotating Shift Work”[All Fields] OR “shift work rotating”[All Fields]))) AND (“Gastroesophageal Reflux”[All Fields] OR “Gastric Acid Reflux”[All Fields] OR “acid reflux gastric”[All Fields] OR “reflux gastric acid”[All Fields] OR “Gastric Acid Reflux Disease”[All Fields] OR “gastro esophageal reflux disease”[All Fields] OR “gastro esophageal reflux disease”[All Fields] OR “Gastro-Esophageal Reflux Diseases”[All Fields] OR (“Gastroesophageal Reflux”[MeSH Terms] OR (“gastroesophageal”[All Fields] AND “reflux”[All Fields]) OR “Gastroesophageal Reflux”[All Fields] OR (“reflux”[All Fields] AND “disease”[All Fields] AND “gastro”[All Fields] AND “esophageal”[All Fields])) OR “gastro esophageal reflux”[All Fields] OR “gastro esophageal reflux”[All Fields] OR “reflux gastro esophageal”[All Fields] OR “Gastroesophageal Reflux Disease”[All Fields] OR “GERD”[All Fields] OR “reflux gastroesophageal”[All Fields] OR “Esophageal Reflux”[All Fields] OR “gastro esophageal reflux”[All Fields] OR “gastro esophageal reflux”[All Fields] OR “reflux gastro esophageal”[All Fields]). The strategies used for searching on Embase and Web of Science databases were adapted as appropriate.

### Eligibility criteria

Studies were eligible for proceeding in the selection process if they contained the following 3 elements, [1] no limitation of participants; [2] exposure to shift work; and [3] assessment of GERD.

### Study selection process

Firstly, the title and abstract of the eligible articles were examined by two researchers (T-YH and *P*-SW) separately. Afterward, full-text screening was applied to check if each study met the inclusion criteria, or those with unclear eligibility in abstract screening. Once an article was considered not to meet the inclusion criteria, three researchers (H-YC, F-JL, and H-CH) comprehensively examineed the article to decide whether to exclude it.

### Data collection

The study characteristics of each selected article, shift work, GERD cases, and the association between shift work and possible GERD were extracted for further analysis. The corresponding authors will be contacted for clarification if the information in the study is not clear.

### Study characteristics

The information on the study characteristics, including publication year, the study conducted country, sample size, the characteristics of participants, and odds ratio (OR) of GERD between shift workers and non-shift groups were collected from selected articles.

### Shift work

In this study, shift work has been defined as “work in irregular working daytime hours”, including evening shift, night shift, on-call shift, or rotating shift (1, 16–18).

### Possible gastro-esophageal reflux disease (GERD)

In this study, possible GERD is including erosive esophagitis and gastroesophageal reflux disease or its associated symptoms, assessed by questionnaire or esophagogastroduodenoscopy (EGD) in each study.

### Statistical analysis

All prevalence ORs for possible GERD were calculated in the shift worker group and non-shift worker group, and the standard error (SE) for the OR were accessed according to the 95% confidence interval (CI) for the OR. The pooled OR and its SE were applied for conducting meta-analysis. In the main analysis, the estimated pooled prevalence OR and its 95% CI were calculated according to the main prevalence ORs and the SEs through a fixed-effects model. The analytical results show the possibility of heterogeneity in ORs extracted from study characteristics of the eligible articles by the fixed effect model (19). Among-study heterogeneity was expressed by means of *I*^2^, in addition to that, the funnel plot was applied to study the publication bias. The subgroup meta-analysis of the shift style, night shift or rotating shift, and gender difference, men only vs. women and men combined were also conducted in this study, and all the analyses were conducted according to Review Manager version 5.4 and R version 3.5.2.

## Results

### Selected studies

**Figure 1:**
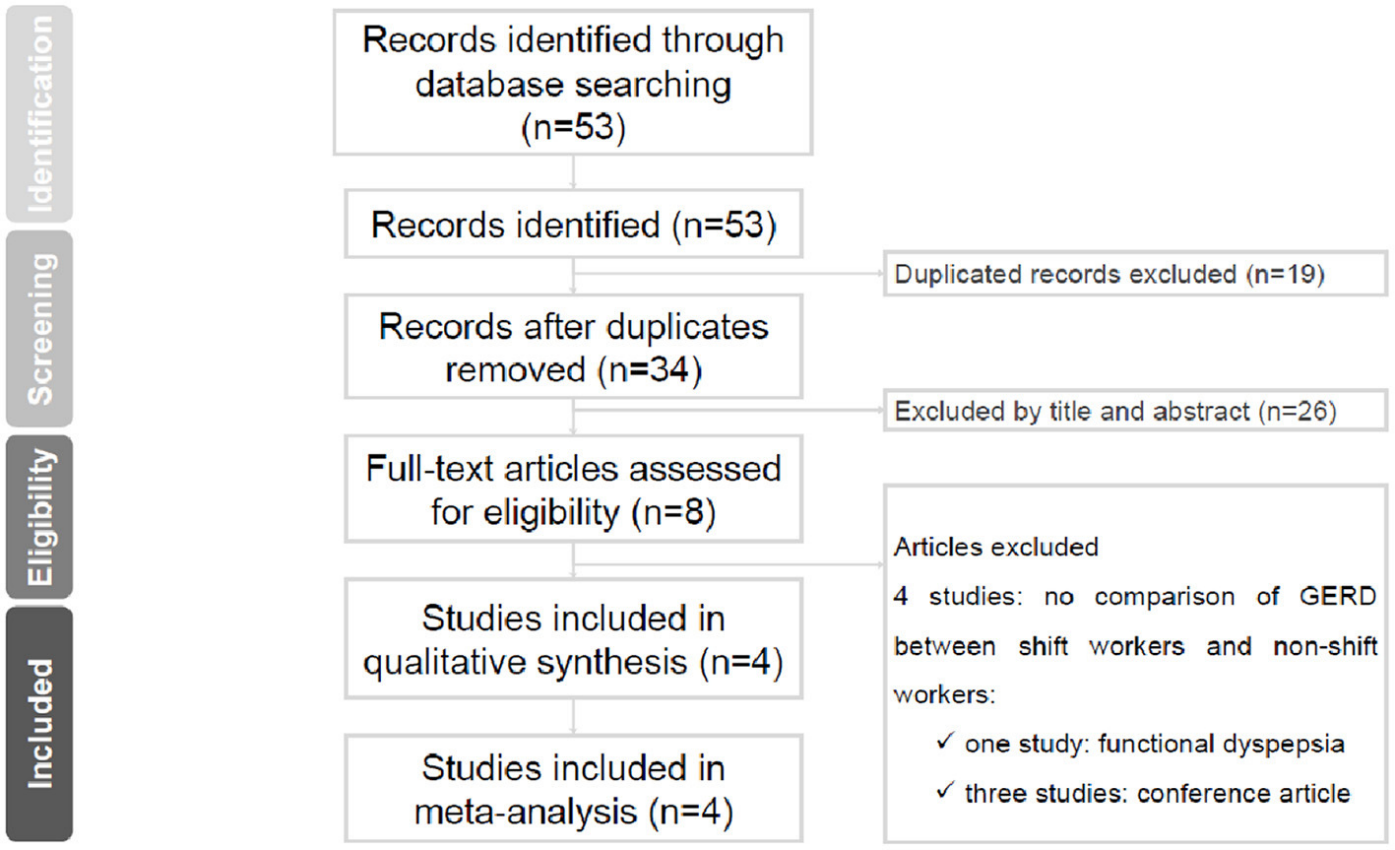
Preferred reporting items for systematic reviews and meta-analyses flow chart.

Figure 1 has shown the selection process of this research by the PRISMA flow diagram. In the initial search stage, 53 articles were identified in three datasets (Pubmed, EMBase, and Web of Science), and 19 duplicates were removed. After screening the abstracts of the remaining articles, 26 articles were excluded. Afterward, through the full-article screening, four records were excluded, for the content does not meet the criteria, no comparison of GERD between shift work and non-shift work exposure. Finally, four studies were recruited in the qualitative survey and the meta-analysis.

### Study characteristics

Table 1 demonstrates the characteristics of the four studies that met our inclusion criteria (20–23), and these studies are all cross-sectional studies. The four cross-sectional studies included Chung et al. (6,040 shipyard workers) (21), Li et al. (15,283 employed patients) (20), Najafimehr et al. (3,590 auto factory employees) (22), and Xue et al. (2,027 workers) (23). Assessment of GERD in three studies (20, 22, 23) was conducted by questionnaire, while the other one (21) was by esophagogastroduodenoscopy (EGD). Two studies (21, 22) illustrated the OR for men only, while the other two showed the OR for both sexes (20, 23). Furthermore, two studies (20, 21) showed night shift concerning GERD, and the others (22, 23) investigated the impact of rotating shift work.

**Table 1:** Studies included in the meta-analysis (*N* = 4).

**First author (year), country**	**Study design**	***N***	**Recruitment**	**Participants**	**Sex**	**Exposure variable**	**Outcome measures**	**Comparison**	**Source**
Chung (2016), Korea	Cross-sectional study	6,040	Workplace	Shipyard workers	Men only	Night shift	Esophagoga stroduode noscopy (EGD)	Erosive esophagitis (EE)	Table 2
Li (2008), China	Cross-sectional study	15,283	Hospital	Employed patients	Men and women combined	Night shift	Chinese version of the reflux diagnostic questionnaire (RQD)	RQD≧12	Table 4
Najafimehr (2018), Iran	Cross-sectional study	3,590	Workplace	Auto factory employees	Men only	Rotating shift	Questionnaire	Heartburn and/or acid regurgitation occurring at least weekly	Table 2
Xue (2021), China	Cross-sectional study	2,027	Hospital	Workers	Men and women combined	Rotating shift	Frequency scale for the symptoms of GERD (FSSG)	FSSG> 8	Table 2

*SW*, shift work; EGD, Esophagogastroduodenoscopy; *EE*, Erosive esophagitis; *RQD*, reflux diagnostic questionnaire; *FSSG*, Frequency scale for the symptoms of GERD.

### Results of individual studies

**Figure 2:**
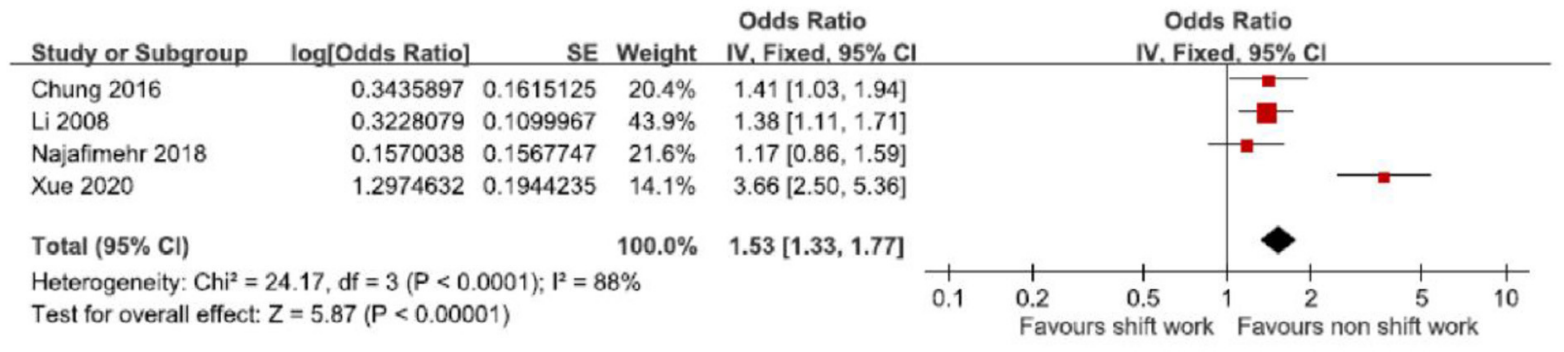
Forest plot of the association between shift work and possible gastro-esophageal reflux disease (GERD) in the 4 studies: a fixed-effect model.

Figure 2 indicates the measures used for examining the association between shift work and GERD. Three articles (20, 21, 23) reported a significant association between shift work and GERD. All four studies (20–23) performed additional or sub-group analysis about OR of GERD according to shift style, night shift work, or rotating shift work.

### Meta-analysis

A fixed-effect model meta-analysis demonstrated the variations in the association between shift work and possible GERD (OR derived from 4 studies) (Table 2, Figure 2) (20–23). In other words, the pooled prevalence has shown a significant positive association between shift work and possible GERD (OR = 1.53; 95% CI 1.33–.77; z = 5.87, *p* = 0.003). The heterogeneity was significant (χ^2^ = 7.26, *p* < 0.00001).

**Table 2:** Measures of the association between shift work and possible GERD used in four studies.

**First author (year/journal), country**	**Sex**	**Comparison**	**OR**	**95% CI (low)**	**95% CI (high)**
Chung (2016), Korea	Men only	Erosive esophagitis (EE)	1.41	1.03	1.94
Li (2008), China	Men and women combined	RQD≧12	1.38	1.11	1.71
Najafimehr (2018), Iran	Men only	Heartburn and/or acid regurgitation occurring at least weekly	1.17	0.86	1.59
Xue (2021), China	Men and women combined	FSSG> 8	3.66	2.50	5.36

*OR*, odds ratio; *GERD*, gastroesophageal reflux disease; *CI*, confidence interval.

**Figure 3:**
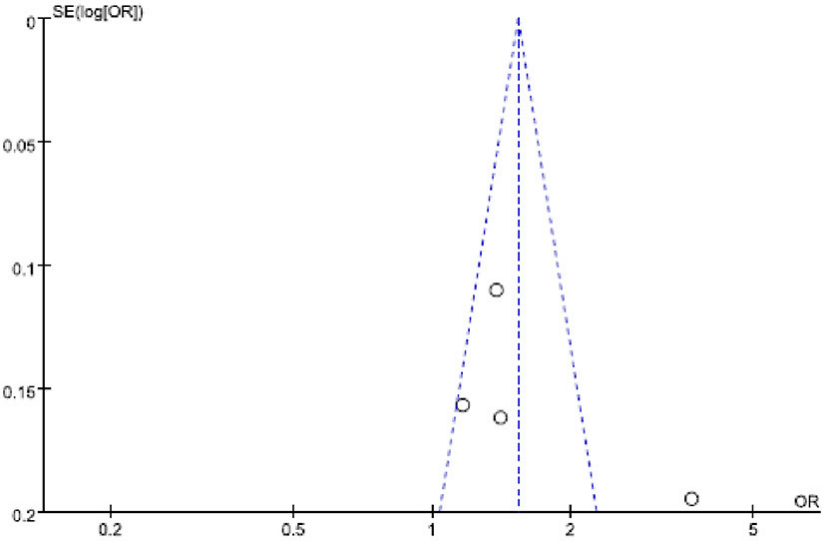
Funnel plot of log-transformed odds ratio of shift work and possible GERD, and standard errors for the 4 studies.

A funnel plot of the log-transformed OR of possible GERD associated with shift work and the SEs among the four OR revealed a relatively smaller number of studies with relatively small SE (i.e., adequate sizes) reporting significant ORs (Figure 3).

### Subgroup analysis

**Figure 4:**
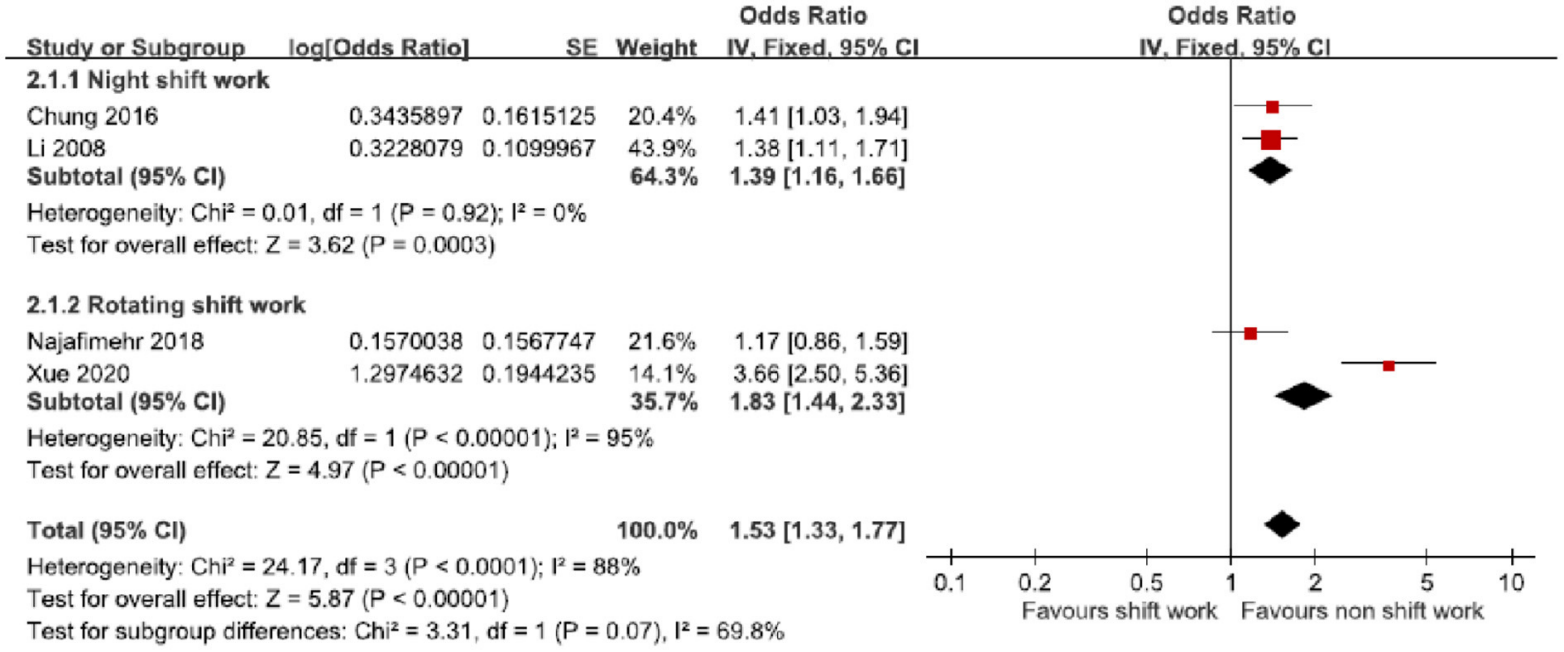
Subgroup analysis of odds ratios of possible GERD based on the style of shift work.

The subgroup analysis of the style of shift work was conducted by fixed-effects model meta-analysis of pooled prevalence ORs (Table 3, Figure 4). In the night shift work group (2 ORs derived from 2 studies) (20, 21), the pooled prevalence OR 1.39 (95% CI 1.16–1.66, *p* = 0.003) was significant. There was non-significant heterogeneity (*I*^2^ = 0%, χ^2^ = 0.01, *p* = 0.92). On the other hand, the pooled prevalence OR of the rotating shift work group (2 ORs derived from 2 studies) (22, 23) 1.83 (95% CI 1.44–2.33, *p* < 0.00001) was significant. There was significant heterogeneity (*I*^2^ = 95%, χ^2^ = 20.85, *p* < 0.00001).

**Table 3:** Subgroup analysis of odds ratio based on whether the participants were rotating shift or night shift.

**Subgroup**	**Odds ratio**	**95% Confidence interval**
**Study participants**		
**Night shift**		
Chung (2016), Korea	1.41	1.03–1.94
Li (2008), China	1.38	1.11–1.71
Subtotal	1.39	1.16–1.66
**Rotating shift**		
Najafimehr (2018), Iran	1.17	0.86–1.59
Xue (2021), China	3.66	2.50–5.36
Subtotal	1.83	1.44–2.33

**Figure 5:**
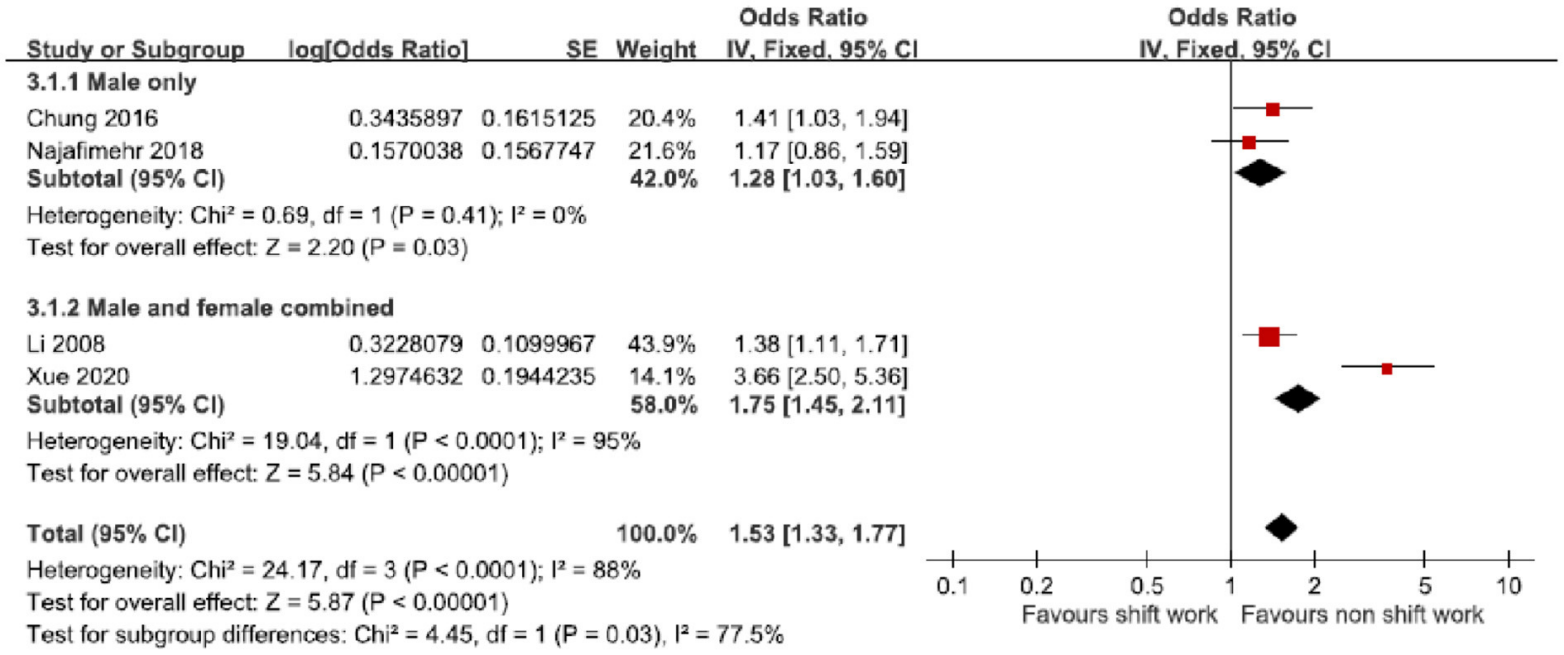
Subgroup analysis of odds ratios of possible GERD based on different genders.

The subgroup analysis of gender difference was conducted by fixed-effects model meta-analysis of pooled prevalence ORs (Table 4, Figure 5). In the men-only group (2 ORs derived from 2 studies) (21, 22), the pooled prevalence OR 1.28 (95% CI 1.03–1.60, *p* = 0.03) was significant. There was non-significant heterogeneity (*I*^2^ = 0%, χ^2^ = 0.69, *p* = 0.41). On the other hand, the pooled prevalence OR of the men and women combined group (2 ORs derived from 2 studies) (20, 23) 1.75 (95% CI 1.45–2.11, *p* < 0.00001) was significant. There was significant heterogeneity (*I*^2^ = 95%, χ^2^ = 19.04, *p* < 0.00001).

**Table 4:** Subgroup analysis of odds ratio based on whether the participants were male only vs. male and female combined.

**Subgroup**	**Odds ratio**	**95% Confidence interval**
**Study participants**		
**Male only**		
Chung (2016), Korea	1.41	1.03–1.94
Najafimehr (2018), Iran	1.17	0.86–1.59
Subtotal	1.28	1.03–1.60
**Male and female combined**		
Li (2008), China	1.38	1.11–1.71
Xue (2021), China	3.66	2.50–5.36
Subtotal	1.75	1.45–2.11

## Discussion

Lifestyle and working environments are important factors of numerous diseases, and GERD is a high-prevalence gastroenterology disorder disease worldwide, as it is stated to affect up to 10–38% of the western adult population more than once a week. To date, literature has supported GERD being strongly associated with several factors that are related to diet, gender, and weight but whether it has an association with working environment still remains unclear. Therefore, this study aimed to work on the relationship between shift work and possible GERD, demonstrated by using the meta-analysis method which is the first meta-analysis study that exam the relationship between possible GERD and shift work. This study focused on analyzing four of the cross-sectional study results and concluded all the results (20–23). However, the focused independent variables in four of the studies were slightly different

Chung et al. (21) focused on comparing the rate of erosive esophagitis between night-shift and non-night-shift participants from South Korea. As it was pointed out that erosive esophagitis is a complication of GERD, which can be more accurately detected, the statistical analysis results showed night shift work is a risk factor for both erosive esophagitis and GERD (21). Li et al. (20) investigated China, and the study focused on finding the differences between the non-GERD patients and GERD patients in age, shift time, diets, working burden, symptoms, and other diseases. The result shows shift time, diet, working burden and stress, material status, and some disease history e.g., pharyngolaryngitis were all independent risk factors associated with possible GERD in China (20). Similar finding has also been observed on the Xue et al. (23) study, reported various information on GERD patients in China. In the study age, shift time, body mass index (BMI), and symptoms were the focused factors and the result shown the risk of possible GERD symptoms is independently associated with night shift work (23). Moreover, in an Iranian study, Najafimer et al. (22) compared the GERD prevalence in rotatory shift and fixed shift GERD patients (22). The result showed rotatory shift has a higher possible GERD prevalence, as it reported 91.6% of the patients were from the rotatory shift group (22). Overall, four of the studies have all shown that the night shift or rotatory shift is a risk factor for possible GERD, furthermore based on four of the analyzed studies, the participating population has all shown to be from the south-east Asian area, which raises the question of whether GERD symptoms are more common among the Asian population. To date, no study has fully addressed this, and it is suggested that more studies should be conducted regarding this issue in future literature in the field.

Compared to non-shift workers, the subgroup analysis indicates the significant positive associations between the night shift and GERD (OR 1.39, 95% CI 1.16–1.66), as well as the rotating shift and GERD (OR 1.83, 95% CI 1.44–2.33). Chung et al. (21) and Xue et al. (23) pointed out night shift workers are related to GERD due to the disrupted circadian rhythm and the circulating melatonin level. Circadian rhythms are constructed based on the molecular circadian clock in the body, which changes our physical, mental, and behavioral actions throughout the day (24). The overall function of the circadian system covered several physical regulations including, hormones secretion (melatonin) gastrointestinal (GI) tract motility, the secretion of digestive enzymes, maintenance of protective mucosal barrier, absorption or metabolism nutrients, cell proliferation, and tissue repair (24, 25). The mismatch or disruptions of circadian rhythms are commonly triggered by two of the factors, sleeping time or eating time, which changes the molecular circadian rhythms; this often occurs in night shift workers and those experiencing jet lag (24, 26). The disruption of circadian rhythms significantly affects the optimal GI function, studies stated long-term circadian rhythms disruption often leads to several GI dysfunctions, including microbiota dysbiosis, traveler's diarrhea, peptic ulcer, inflammatory bowel disease, and GERD (24). As previously mentioned sleeping time is another factors influencing the circadian rhythm, due to the central circadian clock located at the suprachiasmatic nucleus (SCN) regulating the production of melatonin, which is also known as the sleep-promoting hormone. During the disruption of circadian rhythm period, secretion of melatonin is suppressed (24, 27). This has also been mentioned in studies by Konturek et al. (28) and Resuehr et al. (29) where results have shown GERD patients tend to have a lower melatonin level.

In the study by Cheng et al. (30) it been indicated that female shift workers have poorer sleep conditions and more stress than male workers, due to female workers having larger family care burdens (30). However, whether that has a stronger disruption to their circadian rhythms or increases the risk of GERD was not stated. In contrast to male shift workers, female shift workers will have greater stress and poor sleep conditions. This probably has a stronger effect on circadian rhythms leading to a higher risk of GERD, but again there is no study that focused on this field. Thus, the difference in shift work and the prevalence of GERD for both gender groups still remains unevaluated. On the other hand, in Beermann's study which focused on the difference between male and female shift workers, an experiment showed when both genders work under comparable conditions the result showed there was no difference between both genders in psychosocial and physical health (31).

Our subgroup analysis has shown similar trends that the male group (OR 1.28, 95% CI 1.03–1.60) and both genders group (OR 1.75, 95% CI 1.45–2.11) have got a higher GERD risk when they are working in a shift work environment. Among the current literature, there are scant amounts of studies that examine the gender difference in the association between gastrointestinal disease and shift workers. Most studies that investigate the relationship between shift work and gastrointestinal disease only contribute to a specific gender (women ormen). Currently there are only scarce studies that have vaguely pointed out that both genders of shift workers shared a similar risk of having gastrointestinal disease. A meta-analysis study conducted by Wang et al. focused on the relationship between the risk of colorectal cancer and both gender's night shift workers, with given a result of (OR 1.303, 95% CI 1.100–1.544) in women and (OR 1.328, 95% CI 1.039–1.697) in men (15).

Instead of night shift workers, some studies have examined the gender difference. These studies reported that female participants tend to have less severe GERD symptoms, due to women possessing a weaker acid reflux resulting in less severe damage to the esophageal mucosa. In one study, participants with esophageal disease or other diseases that may probably influence their esophageal function were separated into different groups in gender to measure their pH within the esophagus as representing the frequency and level of distal and proximal esophageal acid exposure (32). The experiment results have shown that female participants had a lower esophageal acid exposure. However, the limitation of that study was the huge BMI difference between the participants. In the study, there were 69 female participants with a mean BMI of 30.4 and a standard deviation of 7.8, while a total of 67 male participants had a mean BMI of 29 with a standard deviation of 6.3 (32). According to Hampel et al. (33) and Chung et al. (21), BMI and obesity are associated with the presence of erosive esophagitis due to the larger intra-abdominal pressure followed by more esophageal acid exposure. Moreover, there is a study that stated an opposite finding that female GERD patients would have more severe GERD symptoms than male patients, due to earlier disease cognition, and different disease management (34). The result of that is still very debatable due to the limited number of studies that focus on whether women have less or more severe GERD symptoms thanmen; more study is needed to evaluate whether there is a difference between genders.

Last but not the least, there are no studies that investigate the gender difference between night shift workers and GERD risk, therefore it could be a notable research topic in the future to ascretian whether female night shift workers do or do not have a higher GERD risk than male night shift workers. Based on the findings of the aforementioned literatures, this is still a debatable topic, more evidence about possible GERD is needed in sleep conditions and the biological differences between male and female night-shift workers.

In the present study, there were several limitations. First, the common methodological questions with attrition bias, such as health worker effects, that the workers who have possible GERD symptoms might be more likely to quit their shift work job or avoid working shifts, will influence the four included studies, moreover the definition of possible GERD has covered several of symptoms that are difficult to draw a robust conclusion regarding the relationship between shift work and GERD. Second, in the study, the external factors and confounding factors, such as shift work, pre-conditions, and co-morbidities are not taken into consideration. Whether shift work employees are unwilling, rewarded, unpaid, voluntary (or not), could not be identified, and the intensity of shift work was unclear. Moreover, in regard to the issue of confounding factors, the participant's BMI, dietary patterns, age, sex, and sleep apnoea condition are all considered particularly prevalent pre-conditions and co-mobidities of GERD among shift workers however this study could not adjust those factors due to the meta-analysis setting. Third, potential personal confounders or other occupational factors were not considered which could modify the association between shift work and possible GERD risk, such as age, BMI, occupation, as well as diet or substance habits, such as excessive sweets, coffee, spicy foods, smoking or alcohol consumption. Fourth, the meta-analysis articles all concern south-east Asian population participants, which reduce the generalizability of the finding, this finding also gives rise to the issue of the need for addressing possible GERD symptoms in other ethnic groups. Fifth, the outcome measurement methods in four of the analyzed studies were heterogenous, while one of the meta-analysis articles used a self-report questionnaire approach (RQD) that has been stated in the literature to be problematic in the accuracy of the analysis result (35), due to the limited amount of literature in the field, the designed protocol of this study had to encompass this article, for future study other the quantitative GERD study is needed.

## Conclusion

In conclusion, the analysis shows there is an association between the night shift or rotation shift with possible GERD prevalence rate, which fits the current literature finding. In our analysis, both genders shared very similar rates of having possible GERD symptoms. However, in the present study, we have found two factors that may have the potential to show the gender differences in possible GERD prevalence rates. Unfortunately, there are not many studies that focus on the field of gender differences in possible GERD prevalence rate so far, therefore the gender differences in possible GERD prevalence rate remain unevaluated. However, this study has shown a positive association between shift work and possible GERD.

## Data availability statement

The raw data supporting the conclusions of this article will be made available by the authors, without undue reservation.

## Author contributions

Conceptualization, formal analysis, and supervision: C-CY. Methodology and software: H-YC and C-CY. Investigation: H-TC, H-YC, T-YH, P-SW, F-JL, H-CH, C-CY, and C-HK. Writing–original draft preparation: H-TC, T-YH, P-SW, and C-CY. Writing–review and editing: H-YC, C-CY, and C-HK. All authors contributed to the article and approved the submitted version.

## Funding

This work was supported partially by the Research Center for Precision Environmental Medicine, Kaohsiung Medical University, Kaohsiung, Taiwan from the Featured Areas Research Center Program within the framework of the Higher Education Sprout Project by the Ministry of Education (MOE) in Taiwan and by Kaohsiung Medical University Research Center Grant (KMU-TC111A01 and KMUTC111IFSP01), and Kaohsiung Municipal Siaogang Hospital, Kaohsiung Medical University (S-110-03).

## Conflict of interest

The authors declare that the research was conducted in the absence of any commercial or financial relationships that could be construed as a potential conflict of interest.

## Publisher's note

All claims expressed in this article are solely those of the authors and do not necessarily represent those of their affiliated organizations, or those of the publisher, the editors and the reviewers. Any product that may be evaluated in this article, or claim that may be made by its manufacturer, is not guaranteed or endorsed by the publisher.

## Abbreviations

BMI: body mass index
CI: confidence interval
EGD: esophagogastroduodenoscopy
GERD: gastro-esophageal reflux disease
GI: gastrointestinal
OR: odds ratio
PPI: proton pump inhibitor
SCN: suprachiasmatic nucleus
SE: standard error.
